# Bioinspired and Green Synthesis of Silver Nanoparticles for Medical Applications: A Green Perspective

**DOI:** 10.1007/s12010-023-04719-z

**Published:** 2023-09-05

**Authors:** Fareeha Arshad, Gowhar A. Naikoo, Israr U. Hassan, Sai Raghuveer Chava, Mohamed El-Tanani, Alaa A Aljabali, Murtaza M. Tambuwala

**Affiliations:** 1 Department of Mathematics and Sciences, College of Arts and Applied Sciences, Dhofar University, Salalah, PC 211 Oman; 2 College of Engineering, Dhofar University, Salalah, PC 211 Oman; 3 Department of Chemistry, Texas A&M University, Kingsville, TX USA; 4 College of Pharmacy, Ras Al Khaimah Medical and Health Sciences University, Ras Al Khaimah, United Arab Emirates; 5 Department of Pharmaceutics and Pharmaceutical Technology, Yarmouk University, Irbid, 21163 Jordan; 6 Lincoln Medical School, University of Lincoln, Brayford Pool Campus, Lincoln, LN6 7TS UK

**Keywords:** Silver nanoparticles, Green approach, Antibiotics, Plant extract, cancer

## Abstract

Silver nanoparticles (AgNPs) possess unmatched chemical, biological, and physical properties that make them unique compounds as antimicrobial, antifungal, antiviral, and anticancer agents. With the increasing drug resistance, AgNPs serve as promising entities for targeted drug therapy against several bacterial, fungal, and viral components. In addition, AgNPs also serve as successful anticancer agents against several cancers, including breast, prostate, and lung cancers. Several works in recent years have been done towards the development of AgNPs by using plant extracts like flowers, leaves, bark, root, stem, and whole plant parts. The green method of AgNP synthesis thus has several advantages over chemical and physical methods, especially the low cost of synthesis, no toxic byproducts, eco-friendly production pathways, can be easily regenerated, and the bio-reducing potential of plant derived nanoparticles. Furthermore, AgNPs are biocompatible and do not harm normally functioning human or host cells. This review provides an exhaustive overview and potential of green synthesized AgNPs that can be used as antimicrobial, antifungal, antiviral, and anticancer agents. After a brief introduction, we discussed the recent studies on the development of AgNPs from different plant extracts, including leaf parts, seeds, flowers, stems, bark, root, and whole plants. In the following section, we highlighted the different therapeutic actions of AgNPs against various bacteria, fungi, viruses, and cancers, including breast, prostate, and lung cancers. We then highlighted the general mechanism of action of AgNPs. The advantages of the green synthesis method over chemical and physical methods were then discussed in the article. Finally, we concluded the review by providing future perspectives on this promising field in nanotechnology.

## Introduction

Numerous nanomaterials and their derivatives have been developed in the past decade, including but not limited to metal, non-metal, and carbon-based nanomaterials. In recent years, this field has gained widespread attention from the scientific community from different fields [1–5]. The nanomaterials can be developed with varying physical properties such as the diameter and morphology of the particles produced by changing the external reaction conditions and the chemicals utilised during production. However, there are often a particular set of limitations posed to synthesising nanomaterials for specific applications. These include high instability, incomplete knowledge of the basic mechanisms and actions of the developed nanomaterials, possible toxic effects, and further characterisation steps that would need skilled professionals and expensive lab equipment [6]. Also, issues may arise considering the storage and recycling of the synthesised nanomaterials. Therefore, efforts are being made to develop nanomaterials using ‘green synthesis’ methods to overcome such disadvantages posed by the conventional synthesis methods, as discussed in the Table 1.

**Fig. 1:**
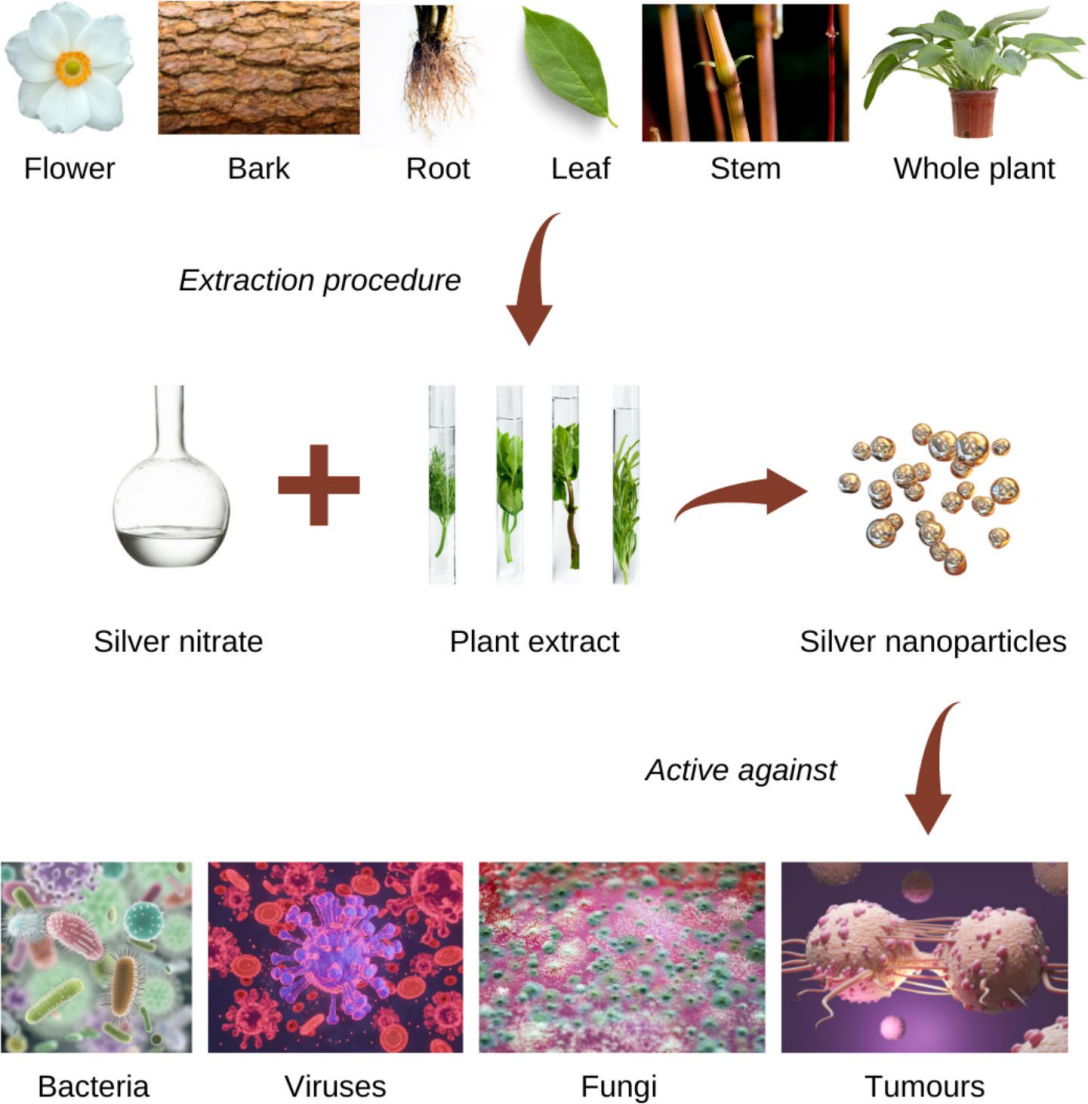
AgNPs derived from plant extracts have shown great potential as antimicrobial, antifungal, antiviral, and anticancer agents

Green synthesis of nanomaterials is an excellent alternative to developing recyclable nanomaterials that are non-toxic through methods that give minimal waste byproducts. Green synthesis methods thus involve eco-friendly methods for nanomaterial synthesis. Recently, multiple biological substrates like bacterial components, fungi, and plant extracts have been employed to synthesise metal and metal oxide based nanomaterials. However, several external parameters like the temperature, pH, pressure, and the chemicals used must be considered before switching to the ‘green method’ of synthesis of nanoparticles. The development of metal and metal oxide-based nanomaterials depends on phytochemicals present in the plant leaves, like carboxylic and ascorbic acids, alkaloids, ketones, flavonoids, aldehydes, tannins, amides, and phenols, among others. These chemicals have the potential to give metal nanoparticles by the reduction of metal salts [6] and also display antioxidant and antibacterial properties (Fig. 1). In addition, these phytochemicals aid in the stability, biocompatibility, and eco-friendliness of the nanoparticles thus developed [7]. Furthermore, plant derived nanoparticles benefit from the bio-reducing agent from the plant sources including the capping agents like phytochemicals that enhance the overall biocompatibility of the synthesized nanoparticles [8]. Green synthesis of nanoparticles also confers additional advantages as compared to the chemical synthesis of nanoparticles including the increase in half-life of the nanomaterial, higher efficiency and therefore are lesser toxic and more biocompatible [9].Therefore, nanomaterial synthesis using a plant source is advantageous because the entire synthesis process is quicker, cheaper, and involves simpler processes than other conventional methods [10].

Among the various metallic nanomaterials based on noble metals, silver nanoparticles are exceptionally unmatched because of their enhanced stability, excellent conductive potential, and catalytic activity. However, the antimicrobial, antifungal, antiviral, and anticancer properties of silver nanoparticles make them superior to the other nanomaterials available. Thus, these silver nanomaterials have widespread applications in biomedical sciences, the food and cosmetic industry, and engineering [11].

In recent years, several works of literature have been published related to the green synthesis aspect of silver nanoparticles, especially the synthesis of nanomaterials from fungi, plants, and bacteria [12–17]. However, to the best of our knowledge, they do not provide an in-depth discussion on the green synthesis of silver nanoparticles using plant extracts and further discussion on their antimicrobial, antifungal, antiviral, and anticancer properties. In this review, we have provided a comprehensive overview of the development of silver nanoparticles, including the leafy parts of the plant, their seeds, flowers, stem, bark, and roots. We then discussed the antimicrobial, antifungal, antiviral, and anticancer properties of the silver nanoparticles, especially in breast, prostate, and lung cancer cases. We then provided a general mechanism of action of silver nanoparticles. Then, we highlighted the advantages of the green synthesis methods over the conventional physical and chemical nanomaterial synthesis methods. Finally, we conclude the review by discussing the future perspectives of silver nanomaterials and their unique properties.

**Table 1:** Difference between chemical and green synthesis of nanoparticles

S. No.	Chemical synthesis of nanoparticles	Green synthesis of nanoparticles
1	Involves complex procedures with expensive starting materials	Involves simple procedures with cheaper alternatives as starting materials
2	Gives toxic or non-degradable byproducts	Does not release any toxic byproducts
3	Requires elevated temperatures	Does not need high temperatures
4	Involves usage of organic solvents and reagents	Involves natural or biological materials
5	Accumulates large quantities of waste	Does not lead to waste accumulation
6	Makes the use of chemical derivatives	Makes the use of biocatalysts
7	Does not have good atom economy	Has a high atom economy
8	May involve high accident risk	Does not involve any accident risk

## Plants Extract Mediated Synthesis of Silver Nanoparticles (AgNPs)

Due to the versatile properties of silver nanoparticles (AgNPs), such as their enhanced chemical properties, high stability, excellent conductivity, they are used in several applications, especially in the biological field, like detecting biomolecules and drug delivery [18]. Also, the inherent antimicrobial properties of AgNPs have been employed to combat antimicrobial resistance [19, 20]. Furthermore, because of the eco-friendly synthesis procedure, ‘green’ synthesis has gained widespread attention from the scientific communities in the past decade. Therefore, several recent works have focused on developing AgNPs using plant extracts, including their leaves, seeds, flowers, stem, bark, roots, and whole plants. The following section, thus, aims to provide a comprehensive overview of the recent developments in plant extract mediated synthesis of AgNPs.

### From leaf

**Fig. 2:**
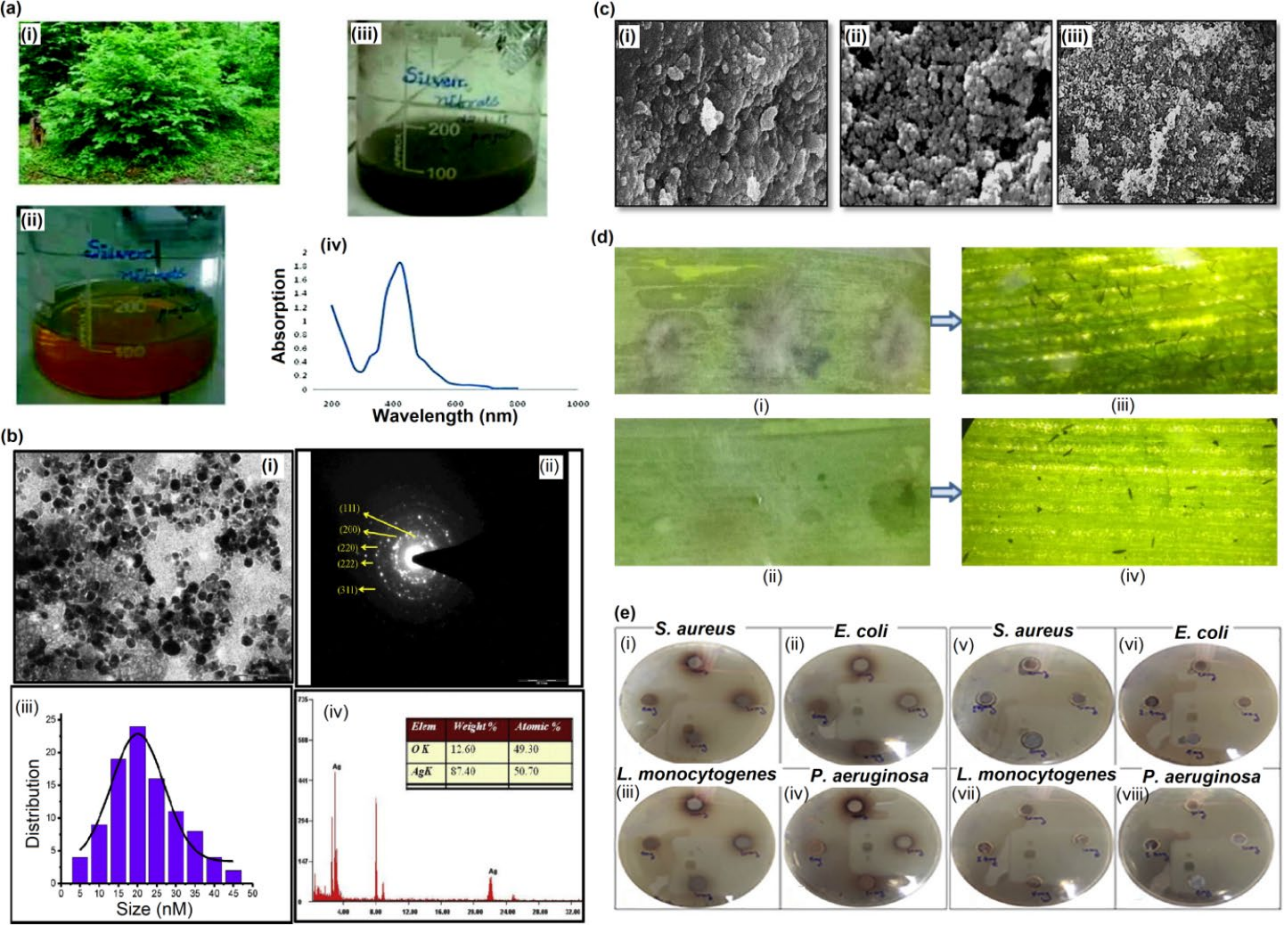
Synthesis of AgNPs using leaf extract. **(a)** Green synthesis of silver NPs from *C. collinus* (i) *Cleisanthuscollinus* plant, (ii) leaves extract, (iii) plant extract reduced silver nitrate solution, (iv) absorption spectra of silver NPs. Adapted with permission from ref. [35], copyright@2020 (Elsevier). **(b)** (i) TEM results confirmed the synthesized AgNPs using *Cestrum nocturnum* was spherical in shape (ii) The diffraction pattern confirmed the formation of metallic AgNPs (iii) The size of AgNPs ranged from 5–45 nm but the average mean size of AgNPs was 20 nm (iv) EDS spectrometer analysis confirmed the presence of silver and copper signals. Adapted with permission from ref. [36], copyright@2020 (Elsevier). **(c)** Comparison of SEM images of metal nanoparticles (i) iron (ii) copper and (iii) silver nanoparticles. Adapted with permission from ref. [34], copyright@2020 (Plos One). **(d)** In vitro inoculation assay of AgNPs against *S. turcica*. (i) Maize leaf inoculated with conidia suspension. (ii) Maize leaf inoculated with conidia suspension and AgNPs. (iii) and (iv) represent microscopic images of the local area of (i) and (ii). Adapted with permission from ref. [26], copyright@2020 (Hindawi). **(e)** Antibacterial activity of AgNPs against *S. aureus*, *E. coli*, *L. monocytogenes*, and *P. aeruginosa*, in different concentrations, where (i), (ii), (iii), and (iv) show AgNPs and (v), (vi), (vii), and (viii) show plant extract. Adapted with permission from ref. [29], copyright@2020 (Heliyon)

Several works report the ‘green’ synthesis of AgNPs from leaf extracts [21–24]. In a 2020 study by Pang and colleagues, AgNPs were synthesized using leafy extracts from *Youngia japonica* plant [25]. About 60 mL of the leaf extract was added to 10 mL of AgNO_3_; the external temperature was maintained at 60℃, and the time taken for the AgNPs synthesis was 40 min. The nanoparticles thus produced had an average size of 20 nm and exhibited a spherical shape. In addition, the authors recorded the antibacterial activity of the NPs produced against the bacteria present on the stem ends of cut lilies. In another study by Huang et al., AgNPs were derived from *Ligustrum lucidum* leaf extract that displayed antibacterial properties against the phytopathogen *Setosphaeria turcica* [26] (Fig. 2). Methods include conidia germination, paper disk diffusion, colony growth, and in vitro inoculation. The authors concluded that the nanomaterials displayed antifungal properties against drug-resistant phytopathogens and thus helped reduce the chemical pesticide usage. In a similar study, Kup and coworkers also developed AgNPs using *Aesculus hippocastanum* leaf extracts and further studied their antibacterial properties. The as-synthesized AgNPs displayed potent antibacterial activity against multiple bacterial strains but lacked antifungal properties [27].

Ramadan and team also successfully derived AgNPs using the leaves of *Melaleuca alternifolia* that displayed successful antimicrobial and antiviral activity against several skin pathogens, including *Staphylococcus aureus*, methicillin-resistant *Staphylococcus aureus*, *Staphylococcus epidermidis*, *Streptococcus pyogenes*, *Klebsiella pneumoniae*, *Pseudomonas aeruginosa*, *Trichophyton mentagrophytes*, *Candida albicans*, herpes simplex virus type 1 (HSV-1), and herpes simplex virus type 2 (HSV-2) [28]. The developed nanoparticles were characterized using the transmission electron microscopy (TEM) technique and were noted to possess a homogenous structure with a spherical shape. The researchers further recorded that the AgNPs displayed an average diameter of 11.56 nm. Likewise, AgNPs have also been successfully derived from the leaves of *Carya illinoinensis* that displayed antibacterial activity against gram-negative bacteria like *E. coli* [21, 29].

In addition, AgNPs synthesized using leafy extracts have been recorded to display antifungal properties. For instance, Nyabola et al. synthesized AgNPs using *Aspilia pluriseta* leaf extracts [30]. They used SEM, TEM, XRD, and dynamic light scattering analyzer (DLS) to analyze the developed nanoparticles further. The authors concluded that the particles had a roughly spherical shape and crystalline structure. Apart from displaying antibacterial activity against *Bacillus subtilis*, *Staphylococcus aureus*, *Escherichia coli*, and *Pseudomonas aeruginosa*, the developed AgNPs also showed antifungal actions against *Candida albicans*. In another study, *Melia azedarach* leaf extracts were used to develop AgNPs [31]. The SEM analyses revealed that the developed AgNPs possessed small, spherical nanoparticles with an average diameter of 23 nm. FTIR spectroscopy further displayed that the hydrolyzable tannic acid in the leafy extract causes the reduction and stability of the nanoparticle produced. In addition, the researchers recorded that the AgNPs demonstrated antifungal properties against *Verticillium dahliae*.

Similarly, multiple recent studies have demonstrated the successful synthesis of AgNPs via green methods using the leaf extracts from *Solanum nigurum* [32], *Curcuma longa* [33], *Syzygium cumini* [34], *Cleistanthus collinus* [35], *Cestrum nocturnum* [36], *Rhizoctonia solani* [37], *Mentha aquatica* [38], that displayed antimicrobial, antifungal, antiviral and anticancer properties.

### From Seeds

In the recent decades, several studies have demonstrated the synthesis of AgNPs using seed extracts of plants *Trigonella foenum-graecum* [39], *Tectona grandis* [40], *Persea americana* [41], *Salvia hispanica* [42], *Phoenix sylvestris* [43], *Phoenix dactylifera* [44], that displayed high antimicrobial, antifungal, antiviral and anticancer properties [45].

In an interesting study, Adnan et al. developed AgNPs using kenaf seed extract, which acted as a bilateral mediator to reduce and cap the Ag^−^ ions under hydrothermal conditions [46]. The TEM microscopy showed that the synthesized AgNPs possessed an average diameter of 7 to 11 nm. Furthermore, the researchers recorded that the AgNPs demonstrated high anticancer activity in the lung cancer cells while remaining non-toxic to the normal cells. In addition, the nanoparticles showed antibacterial activity against both gram-positive and gram-negative bacteria. Also, Qais and colleagues developed AgNPs using the *Carum copticum* extract and were found to possess antimicrobial activity against *P. aeruginosa*, *S. marcescens*, and *C. violaceum* species [47]. Similarly, Zayed and colleagues developed AgNPs using *Pimpinella anisum* seeds that also displayed antimicrobial activity against *Escherichia coli, Staphylococcus aureus, Aspergillus flavus* and *Candida albicans* [48]. Thus, AgNPs can be used to develop therapeutics against bacterial infections.

In another study by Varghese and colleagues, AgNPs were developed using the seed extract of *Trigonella foenum-graecum* [39]. The as-synthesized nanoparticles were roughly spherical, had an average diameter of 33.93 nm, and displayed antibacterial activity against several bacterial strains, including *Staphylococcus aureus*, *Escherichia coli*, and *Klebsiella pneumonia*. In addition, the AgNPs also displayed antifungal activity against *Aspergillus flavus*, *Trichophyton rubrum*, and *Trichoderma viridiae* and anticancer activity against the MCF7 and Vero cell lines.

Similarly, in a study by Hasan et al., AgNPs were synthesized using *Withania coagulans* seeds and were their antibacterial properties were tested against *Staphylococcus aureus* and *Pseudomonas aeruginosa* [49]. It was observed that the as-synthesized nanoparticles allowed for excellent inhabitation potential against the strains via their antioxidant potential. Therefore, AgNPs synthesized from seeds have promising antioxidant and antibacterial potential in a variety of applications.

### From Flowers

In the recent years, flower extracts like *Tagetes erecta* [50], *Nyctanthes arbortristis* [51], *Caesalpinia pulcherrima* [52], *Argemone mexicana*, *Turnera ulmifolia* [53], *Fagonia cretica* [54–56] among many others have also been used to synthesize AgNPs that display antimicrobial, antifungal, antiviral, and anticancer properties. For example, in a recent study, *Chlorophytum borivilianum* was used as a source of reducing agent to develop AgNPs with roughly spherical shape and were well-dispersed with an average diameter of 52 nm [57]. The researchers further recorded that the AgNPs were successful as antimicrobial agents against *Pseudomonas aeruginosa*, *Bacillus subtilis*, Methicillin-resistant *Escherichia coli*, *Staphylococcus aureus*, and *Candida albicans*. In addition, the as-synthesized nanoparticles displayed anticancer activity against the human colon adenocarcinoma cell line. In another similar study, Hariram et al. developed AgNPs from *Tecoma stans* flower extract that functioned as the capping agent during the synthesis process [58]. Furthermore, the AgNPs were functionalized with talc and displayed excellent antimicrobial activity against *Staphylococcus aureus* and *Escherichia coli.*

Bindhu and colleagues also developed AgNPs using *Moringa oleifera* flower extract and studied their antimicrobial properties [59]. The phenol and protein groups present in the extract were responsible for the reduction of AgNPs during the synthesis process. The nanoparticles developed were spherical with a diameter of 8 nm and displayed antimicrobial action against *Klebsiella pneumoniae* and *Staphylococcus aureus*. Similarly, Ajitha and coworkers derived AgNPs from *Syzygium aromaticum* extract that functioned as both capping as well as reducing agents during the synthesis process [60]. The authors added that methoxy and allyl functional groups of eugenols present in the clove extract cause the reduction and stabilization of the AgNPs produced. The resulting nanoparticles displayed antibacterial and antifungal properties and thus demonstrated promising potential in biomedical applications.

### From Stem

**Fig. 3:**
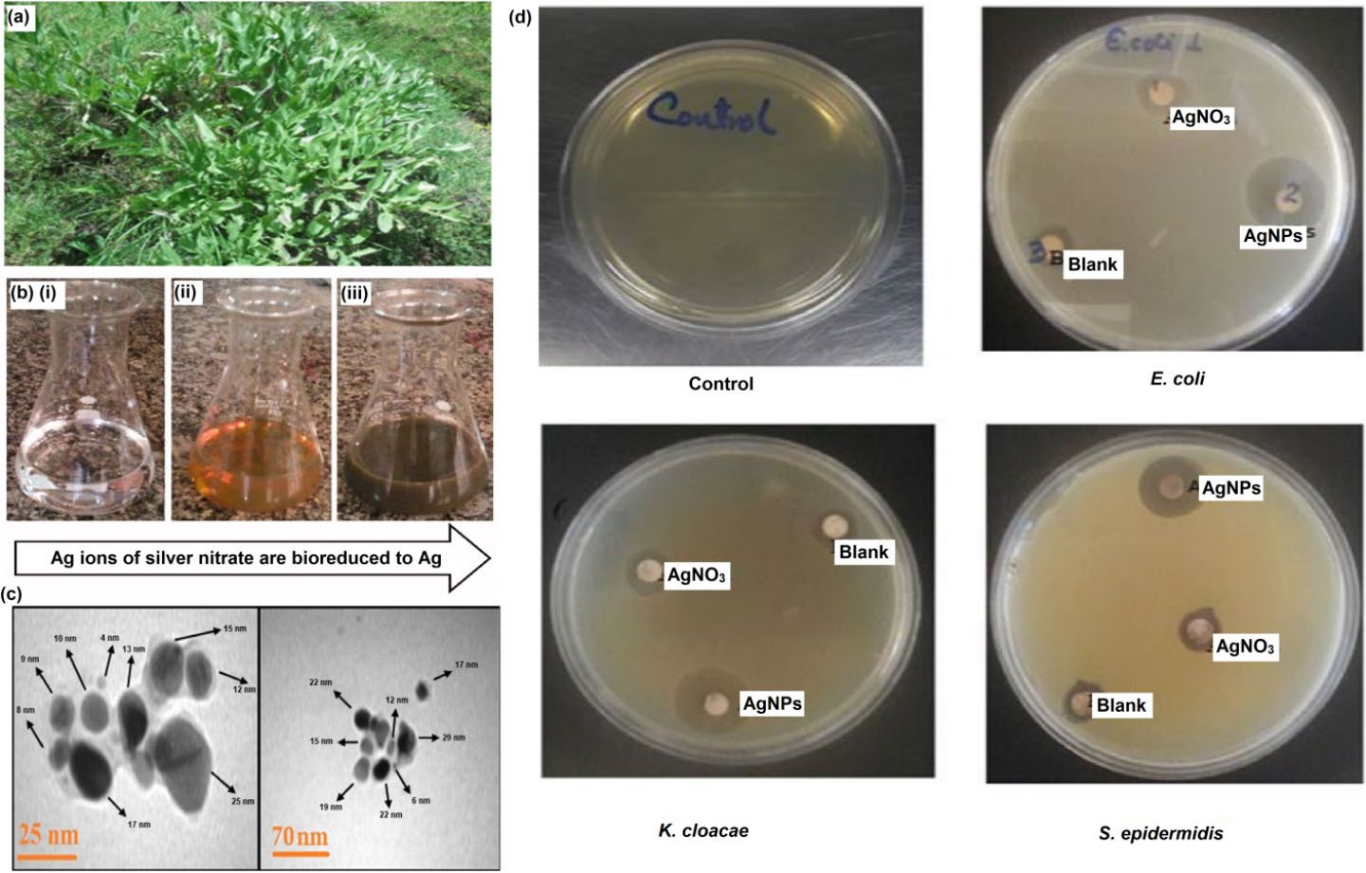
AgNPs synthesis using stem extracts. **(a)** Image *of D. ammoniacum D.* from Sharekord hillside of Iran was taken in May 2017. The plant was used for green synthesis of AgNPs. Adapted with permission from ref. [64], copyright@2018 (IET Research). **(b)** Reduction of silver nitrate through the addition of plant extract that is associated with colour changes. Adapted with permission from ref. [62], copyright@2017 (Taylor and Francis). **(c)** TEM image of biosynthesized silver nanoparticles. Adapted with permission from ref. [66], copyright@2018 (Taylor and Francis). **(d)** Antibacterial activity of the synthesized AgNPs. Adapted with permission from ref. [63], copyright@2017 (Academic Journals)

Several studies in the past decade have successfully demonstrated the synthesis of AgNPs using stem extracts from plants like *Caesalpinia pulcherrima* [61], *Anthemis atropatana* [62], *Moringa oleifera* [63], *Dorema ammoniacum* [64], *Swertia paniculata* [65] and others. In a 2018 study, Khatami and colleagues synthesized AgNPs with an average size of 15 nm from waste dried grass [66] (Fig. 3). These nanoparticles displayed excellent antibacterial activities against *Pseudomonas aeruginosa* and *Acinetobacter baumannii* and antifungal effects against *Fusarium solani*. In addition, the authors also recorded the anticancer activities of AgNPs against Cyclin D1 protein of MCF-7 cell line. Likewise, AgNPs have also been derived using *Garcinia mangostana* stem extract that functioned as the reducing agent during the synthesis process [67]. In another study by Cakić and team, AgNPs were developed using the stem of *Fumaria officinalis* that functioned as the capping and reducing agent [68]. The nanoparticles thus derived displayed antimicrobial function against *Staphylococcus aureus*, *Bacillus cereus*, *Bacillusluteus* in haus strain, *Bacillus subtilis*, *Listeria monocytogenes*, *Escherichia coli*, *Pseudomonas aeruginosa*, *Klebsiella pneumoniae*, *Proteus vulgaris*. They also recorded antifungal activity against *Candida albicans*.

### From Bark

Similarly, the bark of the plant is also used to synthesize AgNPs via the green synthesis method. Extracts of *Zingiber officinale* [69], *Butea monosperma* [70], *Diospyros montana* [71], *Syzygium cumini* [72], among others, have been used to derive AgNPs in the recent decade. For instance, Tormena et al. developed spherical AgNPs with about 13 nm diameter using *Handroanthus impetiginosus* extract, which was capping and reducing agent during the process [73]. The nanoparticles displayed considerable antibacterial activity against the *Staphylococcus aureus* and *Escherichia coli* strains. In a recent study published by Ramzan and team, AgNPs were derived using wild ginger extracts (*Zingiber zerumbet*) that displayed antibacterial activity against multidrug-resistant bacterial strains including *Staphylococcus aureus*, *Streptococcus mutans*, and *Enterococcus faecalis* [74]. Likewise, in a study by Rohaizad and colleagues, the dried bark extract of *Catharanthus roseus* was used to derive AgNPs [75]. The developed AgNPs were functionalized with graphene oxide and displayed particle sizes ranging between 1 and 26 nm. In addition, the epoxy, hydroxyl, and carboxylic groups in the graphene oxide conferred anionic properties to the developed nanoparticles. Also, the AgNPs provided a large surface area for the nanocomposite. The developed nanocomposite thus held the promising potential for antibacterial, antifungal, antiviral, and anticancer functionalities.

### From Roots

**Fig. 4:**
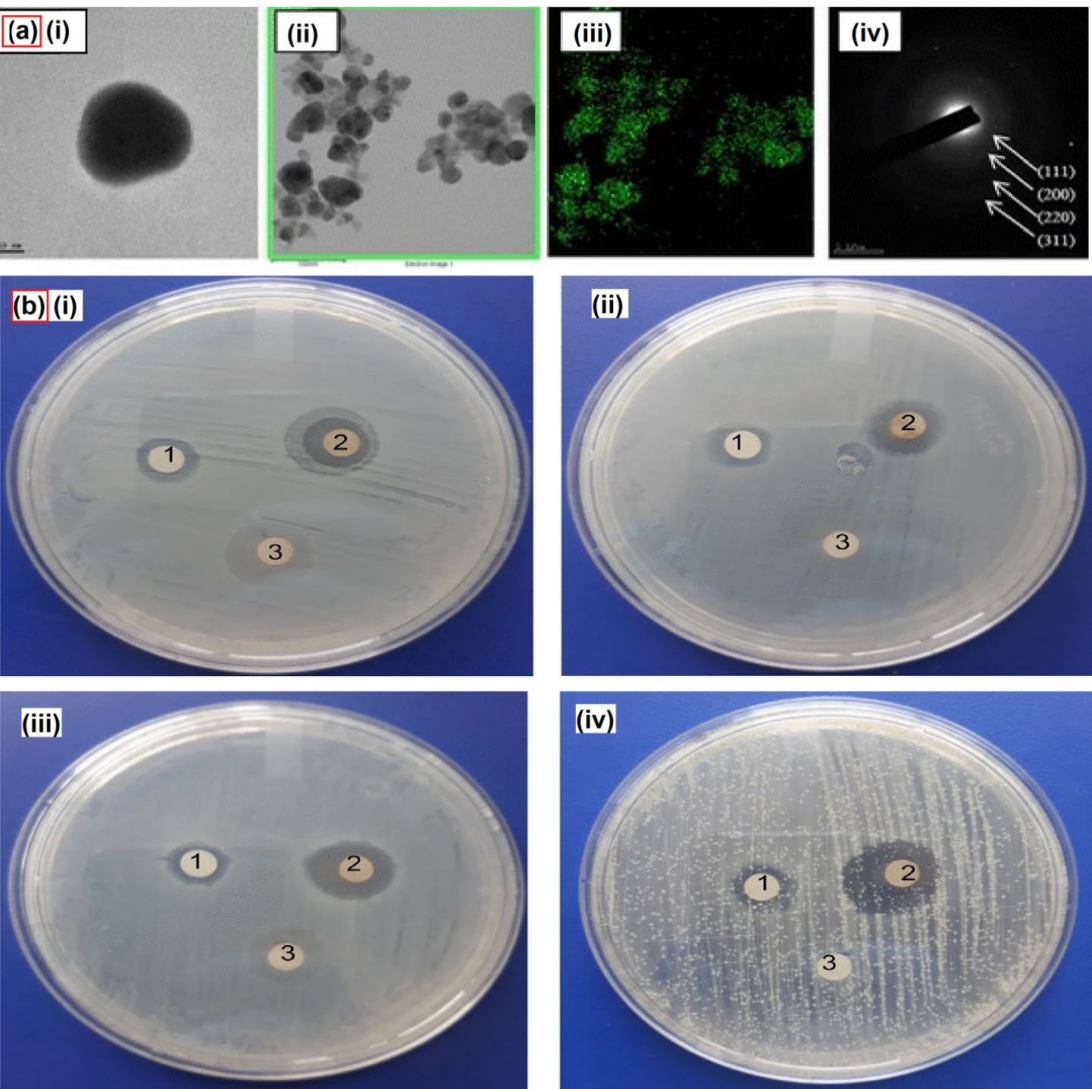
AgNPs synthesis using root extracts. **(a)** (i) FE-TEM images of AgNPs (ii) and (iii) Elemental distribution of AgNPs (iv) SAED patterns of AgNPs. Adapted with permission from ref. [77], copyright@2017 (Springer). **(b)** Antimicrobial susceptibility disk diffusion method. Zones of inhibition of chemical nanoparticles (1), biogenic nanoparticles (2) and aqueous extract (3) against the pathogenic strains *E. coli* (i), *P. aeruginosa* (ii), *S. aureus* (iii) and *C. albicans* (iv). Adapted with permission from ref. [82], copyright@2020 (Nature)

Root extracts from plants have also served as the source for AgNPs. Extracts from *Lepidium draba* [76], *Angelica pubescens* [77], *Arctium lappa* [78], *Allium fistulosum* [79], *Phoenix dactylifera* [80], *Asparagus racemosus* [81] and others have been used to synthesize AgNPs. Garibo and colleagues synthesized AgNPs using the extract from *Lysiloma acapulcensis*, which allows for the reduction of silver to AgNPs and boosts its antibacterial property [82] (Fig. 4). The nanoparticles thus developed showed an average size of 5 nm. The antibacterial potency of the AgNPs was successfully tested against *E. coli*, *S. aureus*, *P. aeruginosa*, and *C. albicans*. The authors concluded that the antibacterial feature was much stronger in these nanoparticles developed via the green method as compared to chemically developed AgNPs and maintained low cytotoxicity among normal cells. In another similar study, AgNPs were synthesized using the *Achillea millefolium* extracts and displayed excellent antimicrobial properties against gram-positive bacteria (*Staphylococcus aureus* and *Bacillus subtilis*) and gram-negative bacteria (*Salmonella enterica*, *Escherichia coli*, and *Pseudomonas aeruginosa*) [83]. Thus nanoparticles developed from the root extracts are an excellent source of AgNPs and demonstrate good antimicrobial and antifungal features.

### From the Whole Plant

As discussed above, several parts of the plant, like the leaves, flowers, bark, stem, and roots, are used for AgNP synthesis. These parts, after being collected, are washed thoroughly and rewashed using distilled water. These parts are then dried and ground to make fine powder or used directly to prepare the extract. The planet extracts of varying pH are then added to different solutions of varying silver salt that function as the metal precursor and then heated to synthesize AgNPs [84–87]. Plants like *Lactobacillus acidophilus* [88], *Brassica oleracea* [89], *Arnicae anthodium* [90], *Sida cordifolia* [91], *Sida acuta* [92], among others, have been recently employed to synthesize AgNPs from their extracts.

For instance, Ali and colleagues synthesized AgNPs using the whole plant extract of *Elaeagnus umbellata* [93]. The phytochemicals present in this plant act as the reducing and capping agents during the synthesis process. Spherical nanoparticles of an average size of 40 nm were synthesized that displayed excellent antibacterial properties and were non-toxic to normal cells. The AgNPs killed the bacterial cells by destroying their cellular structure and disrupting the cell matrix. The authors further recorded that both gram-positive (*S. aureus*) and gram-negative (*E. coli*) bacterial cells were killed off with the AgNPs synthesized. Chavan et al. synthesized AgNPs from *Blumea eriantha* extract. The developed nanoparticles were about 50 nm in size and successfully demonstrated antibacterial and anticancer properties [94]. In another study, AgNPs were derived using the ethanolic extract of Rheum ribes that functioned as stabilizing and reducing agents during the synthesis process [95]. The nanoparticles were used against the MDA-MB-231 breast carcinoma cell line, during which the AgNPs showed toxic effects against the cancer cell lines. The AgNPs were also used successfully against gram-positive *Staphylococcus aureus*, Methicillin-resistant *Staphylococcus aureus* and *Bacillus subtilis* and gram-negative *Escherichia coli* bacteria, showing antibacterial properties too. Thus, these results prove that AgNPs have potent properties that make them an ideal candidate to be used as anticancer, antimicrobial, antifungal, and antifungal agents. Further details of the various plant parts used in AgNPs synthesis and their relevant applications have been discussed in the Table 2.

**Table 2:** Details of the various plant parts used for AgNPs synthesis and their potential applications

Plant part used for AgNPs synthesis	Plant species used	Properties displayed	Pathogenic species affected	Reference
Leaf	*Galega officinalis*	Antimicrobial	*Escherichia coli*, *Staphylococcus aureus* and *Pseudomonas syringae*	[21]
	*Ligustrum lucidum*	Antifungal	*Setosphaeria turcica*	[26]
	*Aesculus hippocastanum*	Antibacterial, antioxidant	*Staphylococcus aureus*, *S. epidermidis*, *Listeria monocytogenes*, *Corynebacterium renale*, *Micrococcus luteus, Bacillus subtilis*, *B. cereus*, *Enterococcus faecalis, Pseudomonas aeruginosa*, *P. fluorescens*, *Escherichia coli*, *Enterobacter aerogenes*, *Klebsiella pneumonia*, *Proteus mirabilis, Candida albicans, C. tropicalis, C. krusei*	[27]
	*Melaleuca alternifolia*	Antimicrobial	*Staphylococcus aureus, Staphylococcus aureus, Staphylococcus epidermidis, Streptococcus pyogenes, Klebsiella pneumoniae, Pseudomonas aeruginosa, Trichophyton mentagrophytes, Candida albicans*, herpes simplex virus type 1 and type 2	[28]
	*Carya illinoinensis*	Antibacterial	*Escherichia coli, Pseudomonas aeruginosa, Staphylococcus aureus, Listeria monocytogenes*	[29]
	*Aspilia pluriseta*	Antibacterial, antifungal	*Bacillus subtilis, Staphylococcus aureus, Escherichia coli, Pseudomonas aeruginosa, Candida albicans*	[30]
	*Melia azedarach*	Antifungal	*Verticillium dahliae*	[31]
	*Solanum nigurum*	Antibacterial	*E. coli*	[32]
	*Curcuma longa*	Antimicrobial	*S.aureus, P.aeruginosa, S.pyogenes, C.albicans*	[33]
	*Syzygium cumini*	Antimicrobial, antifungal	*Staphylococcus aureus, Aspergillus flavus, A. parasiticus*	[34]
	*Cestrum nocturnum*	Antibacterial, antioxidant	*Escherichia coli, Enterococcus faecalis, Salmonella typhi*	[36]
Seeds	*Trigonella foenum-graecum*	Antimicrobial, anticancer	*Staphylococcus aureus, Escherichia coli, Klebsiella pneumonia, Aspergillus flavus, Trichophyton rubrum and Trichoderma viridiae*	[39]
	*Tectona grandis*	Antimicrobial	*Staphylococcus aureus, Bacillus cereus, E. coli, Bacillus cereus, Staphylococcus aureus, E. coli*	[40]
	*Persea americana*	Antimicrobial	*E. coli*	[41]
	*Salvia hispanica*	Antibacterial	*E. coli, S. aureus*	[42]
	*Carum copticum*	Anticancer, antimicrobial	*P. aeruginosa, S. marcescens, C. violaceum*	[47]
	*Pimpinella anisum*	Antioxidant, antimicrobial	*Escherichia coli, Staphylococcus aureus, Aspergillus flavus, Candida albicans*	[48]
Flowers	*Fagonia*	Antimicrobial	*Staphylococcus aureus, E. coli*	[54]
	*Fagonia cretica*	Antibacterial	*Proteus vulgaris, Escherichia coli, Klebsiella pneumoniae*	[55]
	*Chlorophytum borivilianum*	Antimicrobial, antioxidant	*Pseudomonas aeruginosa, Bacillus subtilis, Escherichia coli, Staphylococcus aureus, Candida albicans*	[57]
	*Tecoma stans*	Antimicrobial	*Staphylococcus aureus, E. coli*	[58]
	*Moringa oleifera*	Antimicrobial	*Klebsiella pneumoniae, Staphylococcus aureus*	[59]
Stems	*Caesalpinia pulcherrima*	Antimicrobial, antioxidant	*E. coli, S. typhimurium, K. pneumoniae, B. cereus, B. subtilis, S. aureus, C. rubrum*	[61]
	*Anthemis atropatana*	Anticancer	human colon cancer and mouse fibroblast cell line	[62]
	*Dorema ammoniacum*	Antimicrobial	*Bacillus cereus, Staphylococcus aureus, Escherichia coli, Salmonella typhimurium*	[64]
	*Swertia paniculata*	Antimicrobial	*Pseudomonas aeruginosa, Klebsiella pneumoniae*	[65]
	*Garcinia mangostana*	Antimicrobial	*Escherichia coli, Bacillus subtilis*	[67]
	*Fumaria officinalis*	Antimicrobial, antifungal	*Staphylococcus aureus, Bacillus cereus, Bacillusluteus, Bacillus, Listeria monocytogenes, Escherichia coli, Pseudomonas aeruginosa, Klebsiella pneumoniae, Proteus vulgaris, Candida albicans*	[68]
Bark	*Butea monosperma*	Antimicrobial	*Escherichia coli, Staphylococcus aureus, Aspergillus niger*	[70]
	*Diospyros montana*	Antimicrobial	*B. subtilis, S. aureus, E. coli, K. aerogenes*	[71]
	*Syzygium cumini*	Antibacterial	*Bacillus subtilis, Escherichia coli*	[72]
	*Handroanthus impetiginosus*	Antibacterial	*Staphylococcus aureus, Escherichia coli*	[73]
	*Zingiber zerumbet*	Antimicrobial	*Staphylococcus aureus, Streptococcus mutans, Enterococcus faecalis*	[74]
Roots	*Chlorella vulgaris*	Antimicrobial	*Chlorella vulgaris*	[79]
	*Asparagus racemosus*	Antibacterial	*Escherichia coli, Staphylococcus aureus, Bacillus subtilis, Klebsiella pneumonia, Pseudomonas fluorescence, Aeromonas hydrophila, Edwardsiella tarda, Flavobacterium branchiophilum, Yersinia rukeri*	[81]
	*Lysiloma acapulcensis*	Antimicrobial	*E. coli, S. aureus, P. aeruginosa, C. albicans*	[82]
Whole plant	*Sida cordifolia*	Antibacterial	*Aeromonas hydrophila, Pseudomonas fluorescence, Flavobacterium branchiophilum, Edwardsiella tarda and Yersinia rukeri, Escherichia coli, Klebsiella pneumonia, Bacillus subtilis, Staphyloccocus aureus*	[91]
	*Sida acuta*	Antimicrobial	*Escherichia coli, Staphylococcus aureus, Streptococcus faecalis*	[92]
	*Elaeagnus umbellata*	Antibacterial	*S. aureus, E. coli*	[93]
	*Blumea eriantha*	Antioxidant, antibacterial, anticancer	*Staphylococcus aureus, Bacillus subtilis, Bacillus cereus, Escherichia coli*, MCF-7 cells	[94]
	*Rheum ribes*	Anticancer, antimicrobial	*Staphylococcus aureus, Methicillin-resistant Staphylococcus aureus, Bacillus subtilis, E. coli*	[95]

## Antimicrobial, Antifungal, Antiviral and Anticancer Properties of AgNPs

As discussed in the previous section, the green synthesis of AgNPs has a lot of positive impacts, especially while dealing with environmental problems. Also, the AgNPs derived from extracts of different plant parts like their leaves, roots, barks, stems, whole plants, flowers, and seeds, has been shown to possess high antimicrobial, antifungal, antiviral, and anticancer properties. This section aims to elaborate on the antimicrobial, antifungal, antiviral, and anticancer properties of AgNPs and their potential application as therapeutic agents.

### Antimicrobial Properties of AgNPs

With the increasing microbial resistance against commonly used antibiotics and antiseptics, several studies have demonstrated the antimicrobial action of AgNPs. Microbial resistance against antimicrobial drugs, as observed in methicillin-resistant, penicillin-resistant, and sulfonamide-resistant bacterial strains, is now becoming a severe health hazard [96]. Nanoparticles like AgNPs are an effective alternative to overcome microbial drug resistance. Several in vitro studies have demonstrated the effective action of metal-based nanoparticles against microbes [97]. The resultant antibacterial activity is influenced by two factors [6]: the raw material used to derive nanoparticles and the size of the nanoparticles. The shape of the nanoparticles also influences their antibacterial feature. In a study by Tak and colleagues, it was concluded that truncated triangular nanoparticles demonstrate activity owing to their closely packed structure [98]. This, thus, leads to a higher antimicrobial function of the nanoparticles.

The antimicrobial action of AgNPs causes the rupture of the bacterial cell membrane or generates gaps in the outer membrane of bacteria, thereby killing the bacterial cells [99–101]. In some instances, AgNPs interrupt the bacterial enzymatic activities leading to their death [102]. In a literature review by Pelgrift and Friedman, it was recorded that the AgNPs display antimicrobial activity owing to the Ag + present [103]. However, these ions either interfere with the electron transport chain present in the microorganisms or damage the nucleic acids by linking with those biomolecules. Also, in some instances, silver ions interfere with cell division by interfering with DNA replication, thereby leading to the eventual death of the microbe. Furthermore, the authors added that the Ag^+^ causes reactive oxygen species formation, leading to toxicity in the microbes and the host cells, causing cell death. Therefore, AgNPs are promising for their application as antimicrobial agents against various microorganisms.

### Antifungal Properties of AgNPs

**Fig. 5:**
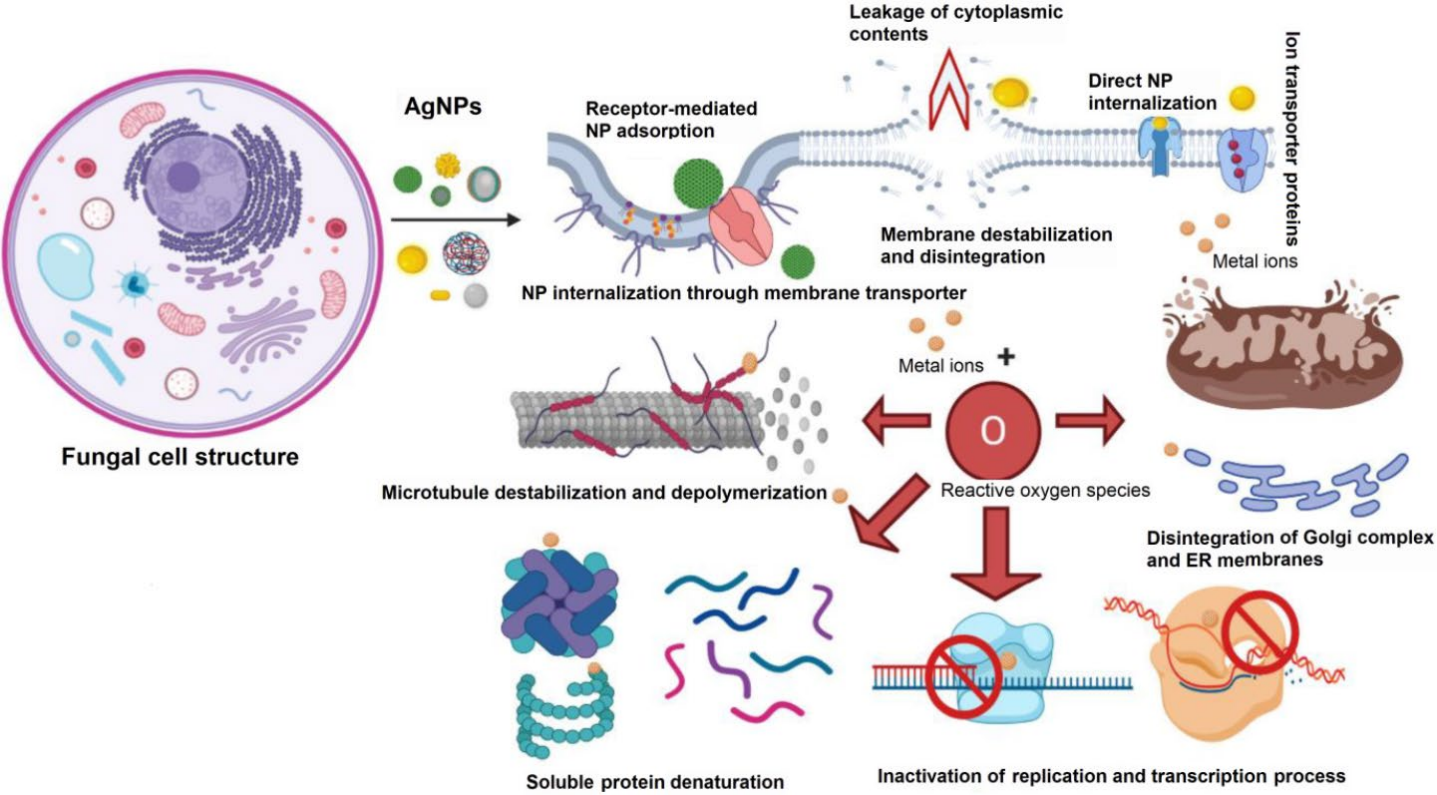
Effect of application of different types of nanoparticles on cellular components and organelles in a fungal cell. Adapted with permission from ref. [104], copyright@2020 (MDPI).

AgNPs have also been shown to have antifungal properties that make them ideal candidates to be used as fungicides against plant pathogens. As discussed previously, the size of nanoparticles influences their mechanical properties and the microstructure [104, 105] (Fig. 5). In addition, the shape of the AgNPs also affects the physicochemical properties: the smaller the nanoparticles, the higher their toxicity [106]. Furthermore, the small-sized of AgNPs mean that they can effectively enter the cells and are more susceptible to oxidative dissolution [107]. Therefore, smaller AgNPs are more effective in generating more significant amounts of silver ions in a short duration.

Several studies confirm the successful antifungal activity of AgNPs against multiple fungal pathogens. AgNPs synthesized from plant extracts of *Brassica oleracea* have shown to display antifungal activities against *Aspergillus* and *Pneumocystis* with its inhibitory effects equivalent to fluconazole [89]. Similarly, extracts from the *Cassia roxburghii* have antifungal effects against A. fumigates, *A. niger*, *A. flavus*, *Candida albicans*, *Penicillium sp.*, *Curvularia sp*., *Rhizoctonia solani*, and *F. oxysporum* [108]. Similarly, extracts from *Diospyros sylvatica* have aided in the AgNP synthesis that displayed inhibitory effects against *Pencillium notatum*, *A. niger*, *C. albicans*, *A. flavus*, *Saccharomyces ceriviseae* [109, 110]. Similarly, AgNPs derived from the extract of *Diospyros paniculata* showed antifungal activity against *C. albicans*, *P. notatum*, *S. ceriviseae*, *A. niger*, *A. flavus* [111]. Garlic plant extracts (*Allium sativum*) based AgNPs have also shown antifungal properties against *Fusarium graminearum* [112]. The researchers recorded that the AgNPs displayed intense activity against the mycelium growth, germinating spores, developing germ tubes, and the toxins the fungi produced, thus inhibiting biofilm formation.

In another recent study by Hussain and colleagues, AgNPs were synthesized from *W. coagulant* fruit extract and were tested for their anti-fungal and anti-bacterial applications for a honeybee pathogens [113]. The study confirmed that AgNPs derived from plant sources show excellent anti-fungal properties against a wide range of pathogens. AgNPs function by disrupting the fungal cell wall, damaging the surface protein and nucleic acid molecules. Also, the nanoparticles accumulate reactive oxygen species within the fungal cell and block the proton pumps [114, 115]. Also, AgNPs aid in the accumulation of silver ions that causes respiration blockage and subsequent damage to the electron transport system, thereby leading to apoptosis [116].

### Antiviral Properties of AgNPs

Several studies in the recent decade have focused on studying the inhibitory effects of AgNPs on multiple virus strains like the feline coronavirus [117], adenovirus [118], herpes simplex virus [119], yellow mosaic virus, dengue virus [120], human immunodeficiency virus (HIV) [121], parainfluenza virus [122], influenza virus, hepatitis B virus, and others [123].

Generally, the AgNPs cause cell death by blocking the replication of the virus particles or interacting with the virus protein layer, blocking their ability to infect the host cells [124]. Lara et al. demonstrated that in the HIV-1 cells, AgNPs function as an inhibiting agent by targeting the gp120 proteins, which hinders its attachment to the host cell membranes [125]. At even low levels of 10 nm sized AgNPs (10–25 µg/ml), the replication of Tacaribe virus particles is inhibited; therefore, no new virus progenies are formed [126]. Xiang et al. studied the effect of AgNPs on the H_3_N_3_ influenza virus and the effects of the nanoparticles on the Hemagglutinin glycoprotein, which is the main protein layer that attaches to the host membrane receptor [127]. The authors recorded that AgNPs interfere with the disulfide bond on the Hemagglutinin glycoprotein molecules and therefore inhibit the entry of the viral genome into the host cells. Likewise, another study on Herpes simplex virus type-1 (HSV-1) revealed that AgNPs capped with mercaptoethane sulfonate at 400 µg/ml cause inhibitory effects on the virus [128]. In addition, the researchers recorded that AgNPs stop the release of new progeny at low concentrations of 6.25 µg/ml and high doses of 100 µg/ml, and the viral replication is completely inhibited.

Studies have also demonstrated that the overall size of the nanoparticles affects their activity against the various viruses. For instance, Gaikwad and colleagues discovered that the size and the zeta potential of AgNPs influence their overall activity against the human parainfluenza virus type3 and herpes simplex virus [122]. Similarly, in another such work, it was discovered that AgNPs attached to the gp120 protein on the virus surface showed inhibitory effects against the HIV-1 virus by interfering with the attachment of virus particles with the host cells [121]. Similar results were observed in another study by Avilala et al., where AgNPs displayed inhibitory effects against new castle viral disease by interacting with the gp120 proteins [129]. AgNPs also inhibit the nucleocapsids of virus particles present within the host cells. In addition, these nanoparticles also interfere with cellular processes like protein synthesis, which causes inhibitory effects on the viruses.

### Anticancer Properties of AgNPs

As discussed in the previous section, green-synthesized AgNPs have demonstrated anticancer activities against various cell lines.

#### AgNPs Against Breast Cancer

AgNPs have also been shown to possess inhibitory effects, specifically against cancer cells. Notably, these nanoparticles interfere with the function of multidrug resistance transporters present in the breast cancer cells [130]. When overexpressed in cancer cells, these transporter proteins cause the tumours to develop resistance against cancer drugs, rendering chemotherapies useless [131]. Also, AgNPs are readily taken up by breast cancer cell lines because these tumours overexpress folate receptors [132]. Therefore, more cytotoxic effects are observed in the breast cancer cells compared to normal cells. In addition, breast cancer cell lines have been shown to have altered surface glycosylation patterns. Soybean agglutinin conjugated nanoparticles, like AgNPs, have enhanced toxicity against cell surface patterns like in MDA-MB-231 and MCF-7 cell lines [133]. On the contrary, the AgNPs have shown negligible activity against non-cancerous cells.

Cell-penetrating peptides are often used along with AgNPs to aid their intracellular transport within the cancer cells [134]. For instance, in a study by Farkhani and colleagues, it was reported that AgNPs functionalized with cell-penetrating peptides enhance the internalization and cell toxicity of the nanoparticles against MCF-7 breast adenocarcinoma cells [135]. In another recent study, Gopisetty showed that AgNPs trigger autophagy in breast cancer cells [130]. Furthermore, several studies suggest reactive oxygen species (ROS) dependent AgNP-induced endoplasmic reticulum stress is an underlying mechanism for overcoming multidrug resistance in breast cancer cell lines [130]. In a recent study by Gomathi et al., tamarind fruit shells were used to synthesize AgNPs [136]. The developed nanoparticles caused apoptosis in human breast cancer cells. According to the authors, the apoptotic action occurred dose-dependent, and ROS were generated that caused mitochondrial and DNA damage in the cancer cells.

**Fig. 6:**
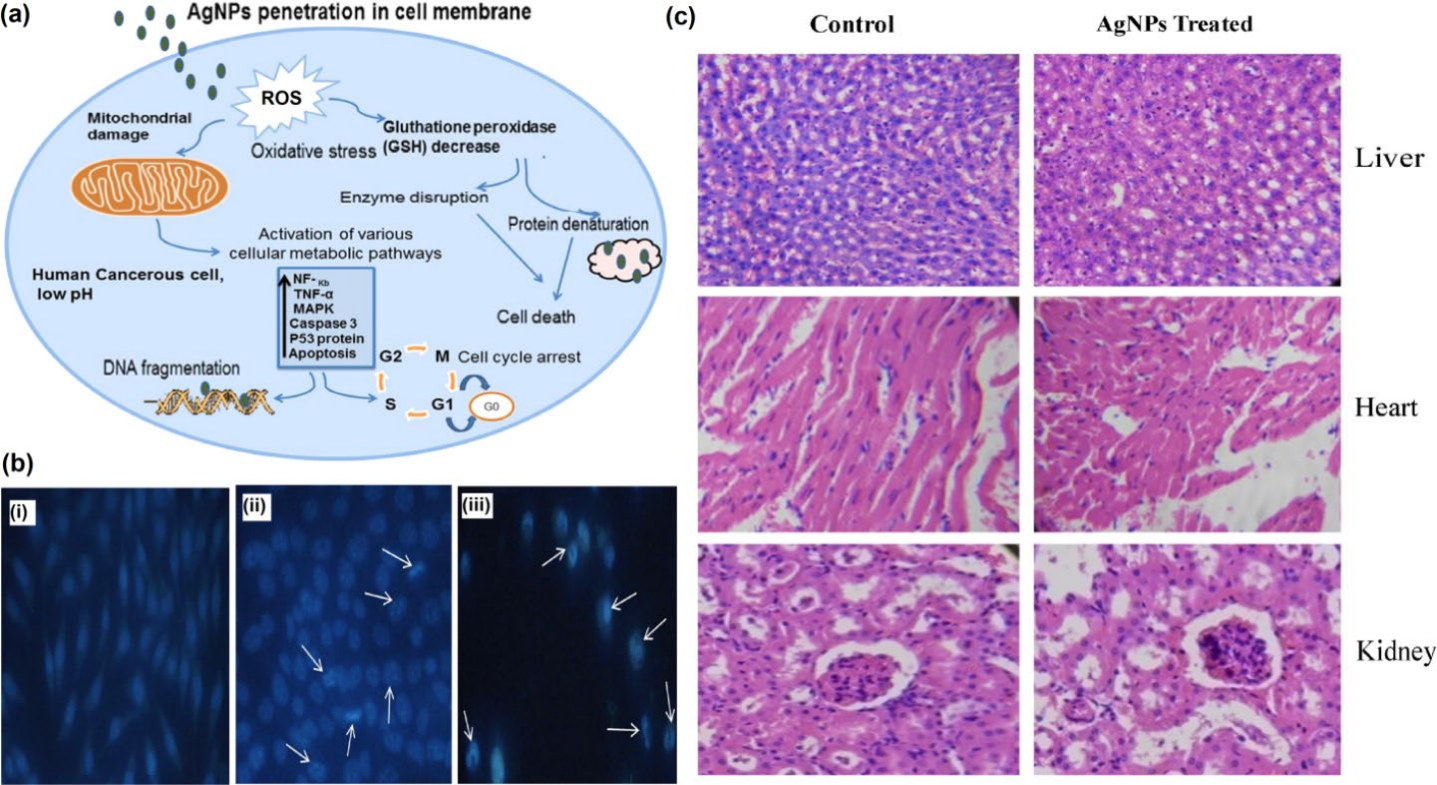
Anticancer activity of green synthesized AgNPs. **(a)** Possible anticancer mechanism of green synthesized AgNPs and various pathways involved in cytotoxic effects of AgNPs. Adapted with permission from ref. [137], copyright@2021 (IOP Science). **(b)** DAPI nuclear staining of (i) control, (ii) cisplatin-treated, and (iii) AgNPs-treated cells. Adapted with permission from ref. [139], copyright@2021 (Springer). **(c)**In vivo toxicity investigation of AgNPs on mice organs such as liver, heart and kidney. Adapted with permission from ref. [140], copyright@2021 (Elsevier)

Apart from apoptotic death in breast cancer cells, AgNPs also cause restriction on transcription of hypoxia-inducible factor-1 (HIF-1) and induces the expression of vascular endothelial growth factor-A (VEGF) [137] (Fig. 6). In a study by Yang et al., AgNPs developed from red amaranth were used against breast cancer cells [138]. The researchers discovered that these nanoparticles caused a reduction in the number and length of blood vessels, and increased toxic effects were recorded in the tumour cells. In addition, it has also been suggested that AgNPs may cause cell toxicity of tumour cells by increasing sub-G1 arrest of cancer cells, triggering cell apoptosis [137].

#### AgNPs Against Prostate Cancer

Green synthesized AgNPs result in apoptosis of prostate cancer cells via the caspase pathway [141]. A recent study by Chen et al. discovered that AgNPs cause activation of HiF-1α, which triggers the AMPK-mTOR pathway and causes apoptosis of PC-3 prostate cancer cells [142]. In another similar study, AgNPs induce ROS-independent autophagy using the same AMPK-mTOR pathway [143]. In another study by Kumari et al., AgNPs derived using *Pinus roxburghii* displayed cell toxicity against prostate cancer cells [144]. The authors recorded that apoptosis was induced via an intrinsic pathway by causing depolarization of the mitochondria and damaging DNA.

AgNPs derived from plant extracts have been shown to display anticancer activity against prostate tumour cell lines. Several recent studies confirm the anticancer activity of AgNPs. For instance, AgNPs synthesized using extracts of *Salvia miltiorrhiza* [145], *Pinus roxburghii* [145], *Alternanthera sessilis* [146], *Dimocarpus Longan Lour* [147], Elderberry and Acai berry [148], *Azadirachta indica* [149], etc. Most studies on prostate cancer cell lines using green synthesized AgNPs, suggest that the nanoparticles cause cell toxicity, ROS generation, and apoptosis by modulating proteins including PARP-1, Bcl2, Bclxl, Bax and Caspase 3 in prostate cancer cell lines [144, 145]. Usually, there is a down-regulation of Stat-3, Survivin and Bcl2 proteins and an up-regulation of Caspase-3 proteins by the AgNPs in the tumour cells [147]. Furthermore, the nanoparticles inhibit prostate cancer cell proliferation, inhibiting tumour growth and leaving the normal cells unaffected.

#### AgNPs Against Lung Cancer

Green synthesized AgNPs derived from plants like *Garcinia mangostana* bark extract [150], *Matricaria chamomilla* extract [151], *Eucalyptus camaldulensis* [152], *Prosopis farcta* [153], *Artemisia oliveriana* [154], *Toxicodendron vernicifluum* [155], *Lonicera Nummulariifolia* [156], *Gossypium hirsutum* [140], *Juniperus chinensis* [139], *Thymus capitatus* [157] have been successfully used against lung cancer cell lines. As discussed earlier, AgNPs usually wipe off the cancer cells via the apoptotic pathway. After the mitochondria release cytochrome c owing to Bax and Bak proteins, the mitochondrial membrane’s permeability increases during the pro-apoptotic event. The AgNPs also enhance the expression of CASP3, CASP9, and miR-192 proteins and decrease the expression of Bcl-2 genes in tumour cells, that in turn causes apoptosis [154, 158].

Fard et al. concluded from their study on AgNPs and their action on lung cancer cells (A549 cell line) that AgNPs display dose-dependent cytotoxicity on the tumour cells and little to no effects on normal cells [154]. Higher toxicity is observed in the A549 cancer cells because mitochondria are more active in tumour cells than the normal cells [159]. Furthermore, the miR-192 decreases the expression of the RB1 gene, which in turn enhances the apoptosis in tumour cells [160]. Studies also demonstrate that silver ions from the AgNPs bind with the DNA and proteins in the cancer cells, thereby hindering their full potential [151].

## General Mechanism of Action of AgNPs

Though the specific mechanism of action for AgNPs is unknown, scientists have studied and recorded the fundamental mode of action for the nanoparticles over the years. Several studies have proposed the possible antimicrobial action against bacterial cells. As AgNPs continuously release silver ions, they can work their antimicrobial action for more prolonged durations [161]. When the AgNPs are taken up by the bacterial cells, the silver ions attach themselves to the cell wall and plasma membrane, affecting their permeability and disrupting the bacterial envelope and causing their denaturation [162]. Upon disrupting the bacterial membrane, the cell organelles also get ruptured, leading to cell lysis [163]. Also, the silver ions cause the deactivation of the respiratory enzymes, leading to ROS accumulation and hindering ATP production [164].

**Fig. 7:**
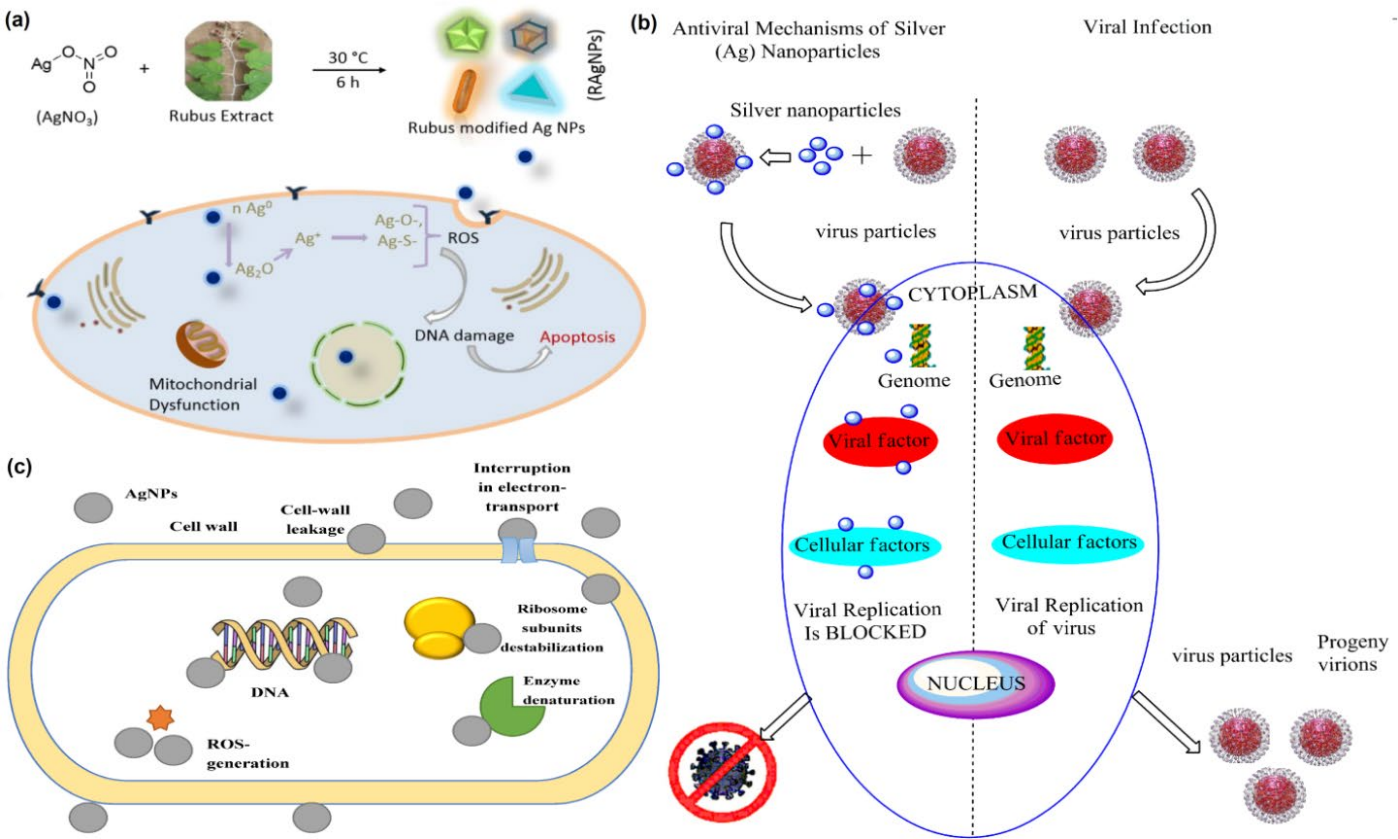
General mechanism of action of AgNPs. **(a)** Representative reaction for AgNPs formation and schematic for nanoparticle induced cancer cell apoptosis. Adapted with permission from ref. [167], copyright@2018 (Nature). **(b)** The proposed possible mechanism of action towards severe acute respiratory syndrome coronavirus 2 (SARS-CoV 2). Adapted with permission from ref. [168], copyright@2020 (MDPI). **(c)** The proposed mechanism of the silver nanoparticles’ influence on bacterial cells. Adapted with permission from ref. [166], copyright@2020 (MDPI).

Furthermore, the synthesis of proteins is affected in the bacterial cells owing to the AgNPs that denature the ribosomes [165]. AgNPs also interfere with the bacterial signal transduction by dephosphorylating tyrosine residues available on peptide surfaces, leading to apoptosis of the bacterial cell and inhibiting the microbial growth, as shown in the Fig. 7 [163, 166]. As discussed previously, the activity of AgNPs is influenced by the morphology and size of the nanoparticles. Owing to the larger surface area of the smaller sized AgNPs, they are more prone to releasing silver ions, thereby displaying higher activity than the nanoparticles with larger diameter. Most studies confirm that AgNPs with size less than 10 nm display higher antibacterial activity than the ones that are larger [163].

Similarly, AgNPs also display antifungal activities against several fungal components. The cell wall of a fungus cell is durable, rugged, and plays a vital role in maintaining cell homeostasis [114]. To overcome such a resistance, green synthesized AgNPs have shown positive activity as antifungal agents [114, 169, 170]. The silver ions aid in the decomposition of the cell wall, damaging the surface proteins and nucleic acid components by releasing ROS and blocking the proton pump [114]. Du and colleagues hypothesized that the rapid influx of AgNPs results in an efflux of intracellular ions that obstructs the electron transport chain [116]. With this, the respiration system within the cell is rendered useless, eventually leading to the apoptosis of the fungal cell. Likewise, AgNPs derived from plant extracts, as discussed in the previous sections, also exhibit antiviral activities. The mechanism of action of the AgNPs against virus cells involves the binding of the silver ions to the outer layer of the virus. This leads to the suppression of the attachment of the virus cells to the host cells. Then the nanoparticles attach with the viral nucleic acid, thereby hindering the virus replication and proliferation within the host cells [168].

Most research hints that anticancer activities occur due to ROS formation [171–173]. After exposure to AgNPs, ROS levels are enhanced, leading to cell toxicity, decreased cell division rate, damage to macromolecules and cell organelles, and eventually cell death [174]. Also, AgNPs interfere with antioxidant molecules present within the cells. This was observed in a study by Miranda, and the team who treated human hepatocarcinoma cells (HepG2) with 10 nm citrate coated AgNPs. The researchers observed a decrease in proteins linked with glutathione metabolism in the cancer cells, while no such changes were observed in the normal cells exposed to the same nanoparticles [175]. Similar results were observed in a study by Barcińska et al., who recorded high ROS levels and lowered antioxidant levels in pancreatic tumour cells upon treatment with AgNPs [176]. The authors further concluded that small-sized AgNPs (2.6 nm) are more cytotoxic than large-sized ones (18 nm). Furthermore, AgNPs also affect other hallmarks of cancers like energy metabolism and drug resistance and affect mitochondrial function and its respiratory chain [167, 177–179]. This, in turn, results in the release of electrons and ROS generation, causing oxidative stress that then interferes with other molecular pathways, finally triggering apoptosis and killing cancer cells [180]. In addition, AGNPs also interfere with the signalling pathways in tumour cells, like activating p53, Caspase-3, LC3-II, ATG7, beclin-1, ATG5, and p-Er K1/2 and deactivating AKT, P62, p-AKT autophagic markers that also results in the programmed cell death of cancer cells [180, 181].

## Advantages of Green Synthesis Over Chemical and Physical Methods

Several studies, as discussed in the previous sections, prove that the green synthesis of AgNPs has been more advantageous than the chemical and physical methods of nanoparticle synthesis. The green synthesis method does not use large amounts of energy or toxic chemicals. Furthermore, the derivation of AgNPs involves inexpensive methods and reagents that are easily accessible. Relatively large amounts of highly stable AgNPs can be derived using plant extracts and do not require high pressure, temperature, or energy conditions and work under moderate operation conditions. Also, plants are easily accessible and safe to handle, which adds to the eco-friendly nature of the method. Because the materials used in the nanoparticle synthesis are biodegradable, the thus derived AgNPs are stable for more extended periods and do not damage the environment. The synthesis process occurs without the production of any significant byproducts and no requirement of strict reaction conditions, thereby reducing the energy consumption during the entire process.

**Fig. 8:**
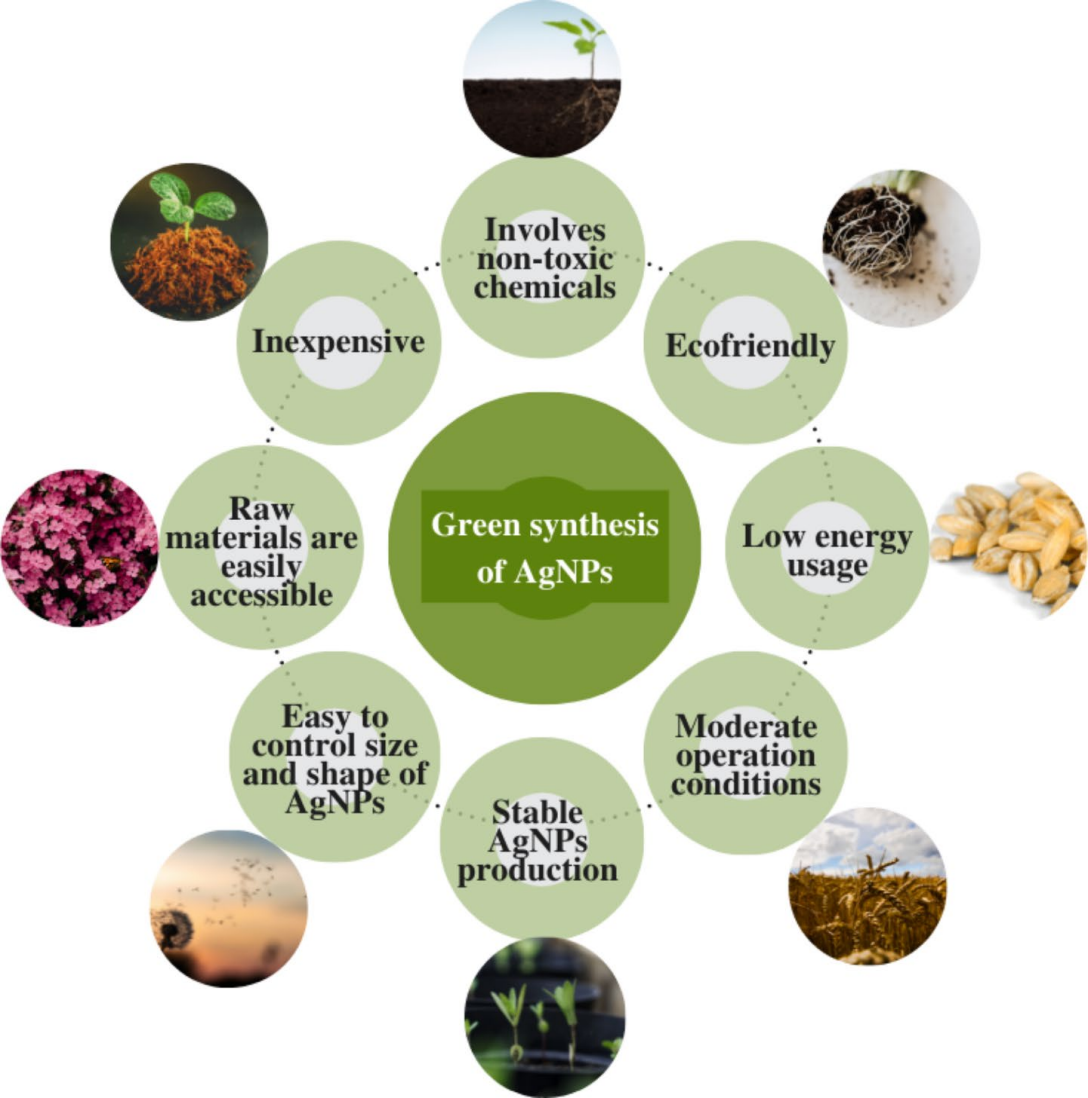
Advantages of green synthesis over chemical and physical methods

Plant-derived AgNPs also possess the potency of plant-based active agents and silver ions, thereby demonstrating more biological activity than AgNPs derived from chemical or physical methods [182]. The plant-based active agents add to the reduction and stabilization of the AgNPs. Multiple biomolecules are available in the plant extracts like phenol groups, quinol, caffeine, polysaccharides, proteins, caffeine, chlorophyll pigments, amino acids, and alcoholic groups that function as the reducing and capping agent during the synthesis process [183]. Phytochemicals like phenols and flavonoids aid in reducing and capping AgNPs, thereby preventing the agglomeration of the developed nanoparticles [183]. Moreover, the bio agents are recyclable and can be regenerated without producing toxic byproducts. Therefore, the plant-derived AgNPs tend to be purer and inherently safer than those developed via chemical and physical methods [184]. In addition, significant variations in AgNPs diameter, morphology, chemical compositions and properties can be developed via the method [185] A brief overview of the advantages of green synthesis as compared to physical and chemical methods have been shown in the Fig. 8.

## Limitations of Green Synthesis of AgNPs

Though green synthesis of nanoparticles is advantageous in many aspects, a few limitations still need to be considered and explored – especially concerning the overall physiochemical aspect of the developed AgNPs. For starters, large-scale green synthesis of AgNPs and fully using the available natural resources is not feasible. Several environmental factors like time constraints, weather conditions, overall plant produce, and availability can also serve as limiting factors that will affect the overall physiochemical properties of the synthesized AgNPs. Secondly, there is limited knowledge that explains the detailed mechanism of action during the green synthesis of nanoparticles. Though the overall role of plant sources in AgNP development is understood, the details behind the reaction kinetics and mechanisms remain to be explored. More importantly, the overall morphology of the nanoparticles derived from different plant sources may vary greatly. Such physical parameters play a crucial role in affecting the overall chemical properties of the green synthesized nanoparticles. Therefore, further research remains to be done to develop highly consistent nanoparticles for their promising application as antifungal, antiviral, and antibacterial agents in various fields.

## Biocompatibility of Silver Nanoparticles

In recent years, several studies have studied and shown the biocompatible properties of AgNPs [186–190]. The overall biocompatibility of AgNPs depends on several parameters, including the surface morphology, diameter, and the charge of the nanoparticles. Therefore, the synthesis method followed for AgNP synthesis greatly influences their biocompatibility. With optimization of the AgNP synthesis pathway and the kind of capping or reducing agents used, the biocompatible properties of the nanoparticles can be tuned to achieve the least toxic exposure and maximize the favourable properties of AgNPs.

Furthermore, AgNPs have unmatched optical and electronic features, including surface-enhanced Raman spectroscopic properties that give them a unique position in developing diagnostic methods for cancer detection [191]. Other factors like the geometry of AgNPs affect the cytotoxic and biocompatible properties; for instance, with decreasing AgNPs size, the cell viability of the nanoparticles increases [192]. Hence under certain conditions, like during inflammatory responses, the toxic properties of the AgNPs increase, which further triggers an enhancement in the secretion of IL-6 and IL-8 [193]. This leads to changes in the confirmation of the albumin properties attached to the nanoparticles that cause scavenger receptor-associated internalization and aids in the immune response [194, 195].

Surface modifications using covalent or non-covalent conjugation methods can also help in the increase in cellular uptake of the AgNPs, both in vitro and in vivo [196]. With the functionalization of the surface morphology of the AgNPs, the stability of the cellular structures can also be affected. For instance, by conferring certain ions on the AgNPs surface, the passive and active absorption of the AgNPs by the cells can be monitored, thereby reducing the overall cytotoxicity of the system and providing the pathways for the development of a highly precise and controlled therapeutic environment.

## Conclusion and Future Prospective

The past decade has seen several collective efforts from the scientific community towards the progressive development of AgNPs using plant extracts. Green synthesis being inexpensive and involving environmentally friendly methods, can be easily scaled up for large-scale production of the nanoparticles. The AgNPs derived via green synthesis routes can be potentially used in multiple biomedical applications, especially as an antibacterial, antifungal, antiviral, and anticancer agents in targeted drug therapy. The nanoparticles do not interfere with the functioning of the normal human cells; they disrupt the activity of tumors, bacterial cells, viral and fungal elements.

However, a few challenges to green synthesis methods still need further work. For instance, the non-uniform sizes of the AgNPs, extensive extraction techniques, and availability of the raw materials are restricted to a particular region. In addition, because of the numerous phytochemicals in the extracts derived from plants, it becomes particularly challenging to monitor their interaction with the nanoparticles developed. Furthermore, several papers demonstrate the successful application of AgNPs as antimicrobial, antifungal, antiviral, and anticancer agents in various laboratory settings, these are still small-scale studies, and their application on a larger scale remains to be explored. Also, because of the laboratory conditions available during the synthesis process of AgNPs and their application as a therapeutic agent, further work still need to be carried out to explore the effects of green synthesized AgNPs in a natural setting outside the laboratory.

More research needs to be done to understand better the chemical composition and concentration of plant extracts for AgNP synthesis. The stability and the physical structure of the developed AgNPs are affected by the chemicals present in the plant extract [32]. Most studies indicate that with increasing capping activity, the diameter of the nanoparticles decreases [86]. Therefore, more studies must be done to understand the interaction of the phytochemicals with AgNPs. Also, with a large concentration of the plant extract, the amount of AgNPs increases [197]. However, this remains true only to a certain level, beyond which large-sized nanoparticles are produced, leading to agglomeration [198].

Regardless of the shortcomings, the AgNPs are highly efficient therapeutic agents against a wide variety of ailments, including the ones caused by microbes, fungi, and viruses. Also, AgNPs have proven high efficiency against multiple cancers like breast, prostate and lung cancers. Thus, green synthesized AgNPs have opened new doors toward the development of drugs and therapeutics against deadly diseases, including those involving multidrug resistance. Further research work in this ever-evolving field, especially towards understanding the mechanism of action of AgNPs against a wide variety of pathogens will help develop better therapeutic strategies against them.

## Data Availability

All data are given in the manuscript.
